# Supplementary material for: Towards unified AI-driven fracture mechanics: the extended deep energy method (XDEM)
Source: Nat Commun. 2026 Aug 15;17:8492. doi: 10.1038/s41467-026-76748-1 (PMC13484471; doi:10.1038/s41467-026-76748-1)
Supplement: Supplementary file 3 [file 41467_2026_76748_MOESM3_ESM.zip › pic/Results/Stress_intensity_factor/Mixed/beta_60/crack_detailed_fields.pdf]

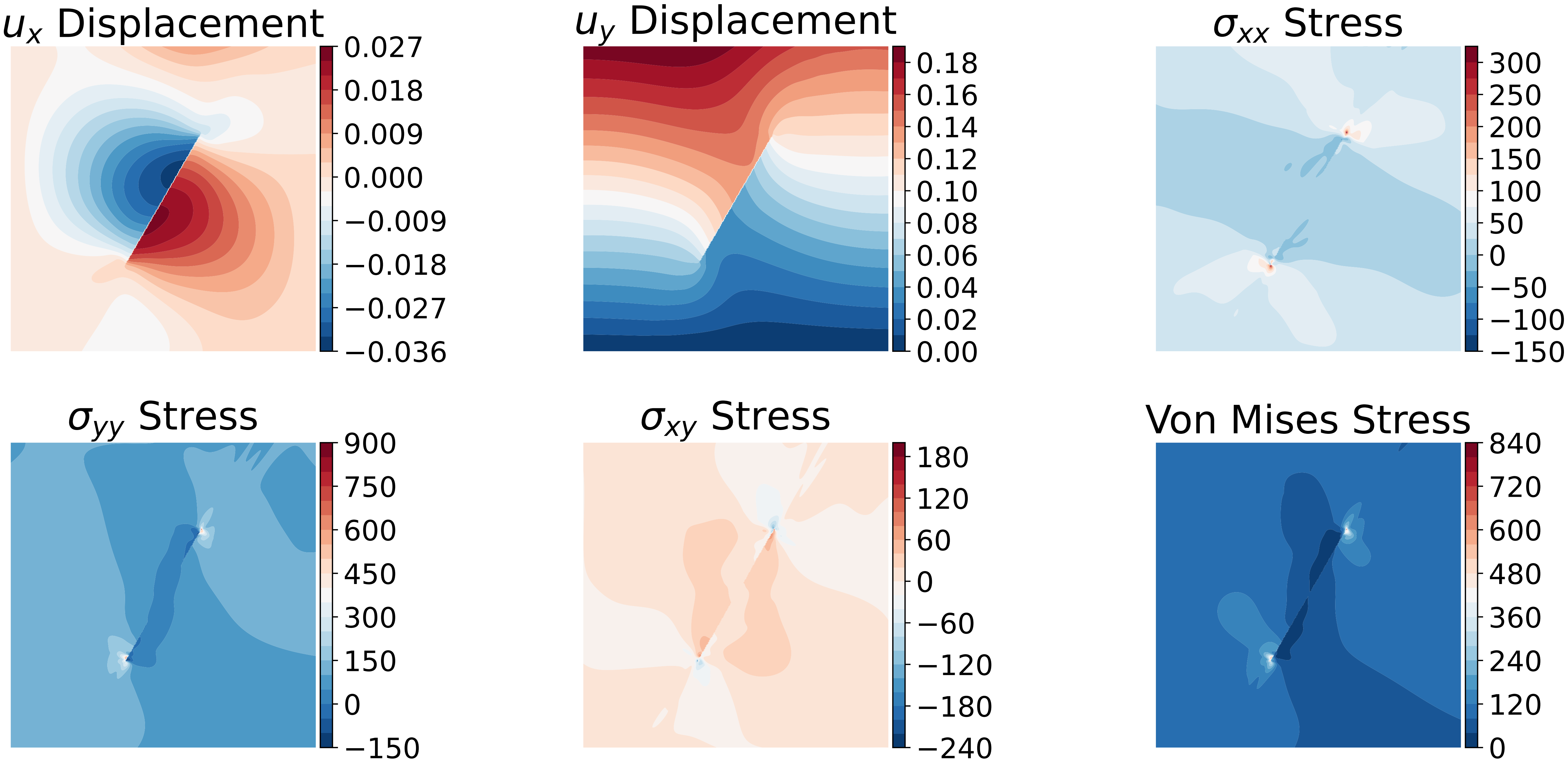

u_x Displacement
0.027
0.018
0.009
0.000
−0.009
−0.018
−0.027
−0.036
u_y Displacement
0.18
0.16
0.14
0.12
0.10
0.08
0.06
0.04
0.02
0.00
σ_{xx} Stress
300
250
200
150
100
50
0
−50
−100
−150
σ_{yy} Stress
900
750
600
450
300
150
0
−150
σ_{xy} Stress
180
120
60
0
−60
−120
−180
−240
Von Mises Stress
840
720
600
480
360
240
120
0
